# All-atom molecular dynamics analysis of multi-peptide systems reproduces peptide solubility in line with experimental observations

**DOI:** 10.1038/srep19479

**Published:** 2016-01-28

**Authors:** Yutaka Kuroda, Atsushi Suenaga, Yuji Sato, Satoshi Kosuda, Makoto Taiji

**Affiliations:** 1 Department of Biotechnology and Life Sciences, Graduate School of Engineering, Tokyo University of Agriculture and Technology, Koganei-shi, Nakamachi, Tokyo 184-8588, Japan; 2 Molecular Profiling Research Center for Drug Discovery (molprof), National Institute of Advanced Industrial Science and Technology (AIST), 2-4-7 Aomi, Koto-ku, Tokyo 135-0064, Japan; 3 Computational Biology Research Core, Quantitative Biology Center (QBiC), RIKEN, IMDA Building (6F), 1-6-5 Minatojima-Minami-machi, Chuo-ku, Kobe, Hyogo 630-0047, Japan

## Abstract

In order to investigate the contribution of individual amino acids to protein and peptide solubility, we carried out 100 ns molecular dynamics (MD) simulations of 10^6^ Å^3^ cubic boxes containing ~3 × 10^4^ water molecules and 27 tetra-peptides regularly positioned at 23 Å from each other and composed of a single amino acid type for all natural amino acids but cysteine and glycine. The calculations were performed using Amber with a standard force field on a special purpose MDGRAPE-3 computer, without introducing any “artificial” hydrophobic interactions. Tetra-peptides composed of I, V, L, M, N, Q, F, W, Y, and H formed large amorphous clusters, and those containing A, P, S, and T formed smaller ones. Tetra-peptides made of D, E, K, and R did not cluster at all. These observations correlated well with experimental solubility tendencies as well as hydrophobicity scales with correlation coefficients of 0.5 to > 0.9. Repulsive Coulomb interactions were dominant in ensuring high solubility, whereas both Coulomb and van der Waals (vdW) energies contributed to the aggregations of low solubility amino acids. Overall, this very first all-atom molecular dynamics simulation of a multi-peptide system appears to reproduce the basic properties of peptide solubility, essentially in line with experimental observations.

Protein solubility, and consequently aggregation, is a critical issue in several areas of biochemical and biopharmaceutical research, such as the production and maintenance of protein pharmaceuticals or industrial enzymes^1^,^2^. However except for general trends, the physico-chemical aspects of protein solubility are not well understood and a hydrophobic/hydrophilic model has been traditionally applied to analyze solubility data. In general, hydrophobic proteins are aggregation prone^3^, whereas proteins displaying charged residues on their surfaces are hydrophilic and thus highly soluble^4^,^5^. However, the initial purpose of the hydrophobic/hydrophilic model was to describe the equilibrium between an amino acid’s solubility in a non-polar and a polar or aqueous environment^6^,^7^.Thus, although this model properly describes the structural stabilization of globular proteins by approximating its interior as non-polar and its exterior as polar^8^, this does not warrant its suitability for describing protein solubility or aggregation tendency.

Experimental scales for describing amino acid’s solubility and aggregation tendencies independently from hydrophobic scales have been reported. Typically, the solubility of individual amino acids have been measured in the 1970’s and standard values for amino acid solubility have been compiled^9^. However, such solubility measurements are subject to artifacts because they are carried out at extremely high peptide (or amino acid) concentrations, which can cause jellification, poor phase separation, sticky fluids, pH shifts, etc^10^.

In order to alleviate issues arising from high molecule’s concentration, two recent studies have measured the contribution of amino acids to the relative solubility change of a model protein using a systematic mutational analysis. In one of them, the solubility of Ribonuclease Sa was examined by systematically mutating Thr76, which is located on its molecular surface, to all of the 20 natural amino acids^11^. In the second example, 5-residue peptide tags, composed of a single amino acid type, were fused to a model protein, a simplified BPTI variant^12^, and the amino acid’s contribution to protein’s solubility was determined by measuring the corresponding BPTI variant’s solubility^13^,^14^. Overall, these two studies suggest that systematic mutational analysis measuring relative solubility changes, could yield a solubility propensity scale, which might provide an estimate for the relative solubility of a poly-peptide from its amino acid sequence alone.

Molecular dynamics (MD) simulation is a powerful method for investigating protein dynamics as well as its interaction with substrates at atomic level^15^,^16^. Recently, it has also been applied to analyzing the molecular interactions at the early stage of self-assembly involving the formation of seed (nucleus) and fibrils in amyloid forming peptides, which are difficult to investigate experimentally^15^,^17^,^18^,^19^. Realistic simulations of multi-peptide systems are computationally demanding and thus time consuming, and implicit solvent^20^ as well as coarse-grained models of amino acids are often used^21^. However, given the key role of peptide-water interaction in the solvation or aggregation of peptides, MD simulations with explicit solvent models are required for characterizing peptide solubility from a genuine physico-chemical view point.

The purpose of this study is to shed light into the molecular determinants of peptide and protein solubility associated to amorphous aggregation (aggregation hereafter) using all atom molecular dynamics (MD) simulations of 10^6^ Å^3^ cubic systems containing approximately 3 × 10^4^ water molecules and 27 tetra-peptides for all 20 amino acids but Gly and Cys. To date, this is a large system for an all-atom MD simulation, and the closest attempt reported so far, is an analysis of amyloidogenic aggregation with systems containing merely 2 to 8 peptides^22^,^23^. The large scale calculation was made possible by intensive use of a state-of-the-art special-purpose computer system for MD simulations^24^, and its scale enabled a systematic and detailed statistical analysis of the results, which strongly suggested that amorphous aggregation properties is quantitatively reproducible using first principle interactions. In particular, the ranking of both the fraction of monomers and the mean cluster size agreed with several, but not all, experimentally derived amino acids’ solubility and hydrophobicity scales. Furthermore, MD simulations indicated that peptides that were soluble at low concentration did aggregate at higher concentration, which is very much in line with experimental observation. Markov state model (MSM) analysis revealed that small clusters containing 2 peptides, which may correspond to seeds, accumulated at the initial stage of aggregation^18^ and then extend to larger ones. Finally, the calculation indicated that high solubility was due to repulsive electrostatic interactions, whereas clusters were stabilized essentially through van der Waals (vdW) interactions, but also accessorily through H-bond and T-stacking interactions. This study is the first of its kind, and it shows that simulations based on solely physico-chemical first principles without introducing artificial parameters can indeed reproduce peptide’s solubility roughly in line with experimental observations.

## Methods

### Model Systems

We performed MD simulations for 18 kinds of tetra-peptides composed of the same four amino acids in this study (e.g. KKKK, RRRR, DDDD, NNNN, etc.), except cysteine and glycine. To remove the effect of the terminal charge and isolate the contribution of the sidechain onto the peptide’s solubility, all the tetra-peptides were capped by an acetyl group (CH_3_CO-) at the N-terminus and N-methyl (-NHCH_3_) group at the C-terminal. Asp and Glu were not protonated (denoted as ASP and GLU in AMBER). On the other hand, we performed the calculation for His both protonated and non-protonated at its epsilon nitrogen (named in AMBER as HIP and HIE, respectively).

The initial configuration was prepared by placing 27 tetra-peptides, with parallel orientation, on a 23.0 Å spaced grid (Fig. S1). Sodium and chloride ions were added using the leap module of Amber 8.0 software package^25^ for neutralizing the net charge of the system. The cubic dimensions, about 10^6^ Å^3^, were chosen to give a peptide concentration of about 40 mM. The total number of water molecules in each system was approximately 30,000^26^.

### MD Simulation

MD simulations were performed using the Amber 8.0 software package^25^ on a personal computer (Xeon 3.2 GHz) equipped with special-purpose computer boards for MD simulations, MDGRAPE-3^27^,^28^. The all-atom point-charge force-field ff99^29^ was used to represent the peptides. All bond-lengths were constrained to equilibrium lengths using the SHAKE module^30^. A 2 fs time integration step was used in all simulations. Long range Coulomb interactions were treated with the Particle Mesh Ewald (PME) method^31^, wherein the real-space component was calculated using MDGRAPE-3, while the host computer calculated the wave number-space component and the bonded-interactions. We used a cut-off radius of 14 Å for the real-space component in order to optimize the balance between the components’ calculation times.

First, energy minimization was performed using the steepest descent protocol, followed by conjugated gradient. After a 5,000 steps energy minimization, the systems were gradually heated from 0 K to 300 K at a heating rate of 6 K/ps. Subsequently, the temperature and pressure were maintained constant at 300 K and 1 atm, respectively, with a coupling constant of 1.0 ps. The MD simulation was performed for 100 ns and data were saved at 10 ps intervals for analysis. Each 100 ns run took approximately 6 months to complete and produced 66 Gbyte of data.

### Analysis of Clusters

The solubility of the peptides was quantitatively characterized by analyzing the formation of clusters (or aggregates) and their sizes in the MD trajectories. In this study, a “cluster” was defined when the distance between two atoms belonging to different tetra-peptide chains was less than the sum of their vdW radius. We defined a “cluster size” (*CS*) as the number of peptides forming a cluster, and calculated a mean cluster size (*MCS*) as *MCS* = (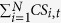) /*N. CS*_*i*, *t*_ means the cluster size to which peptide *i* belongs at time *t*, and *N* is the total number of peptides in the system (27 in this study).

### Energy Calculation

VdW energies and electrostatic energies were calculated using, respectively, the molecular mechanics and generalized Born method^32^, wherein water molecules were replaced with implicit solvent. In the generalized Born calculation, the dielectric constants inside and outside the molecule were set to 1.0 and 78.5, respectively. The H-bonds were calculated with HBPLUS^33^.

### Markov State Model Analysis

We investigated the time dependent cluster’s size distribution using a Markov state model (MSM) and computed a 27 × 27 transition matrix ***T***(*S*_*i*_, *S*_*j*_) describing the transition among cluster size states. The transition matrix was calculated by counting the total number of peptides that undergo the *i* to *j* transition: ***T***(*S*_*i*_, *S*_*j*_) = ∑_*t* = 0_(*S*_*i*,*t*_ → *S*_*j*,*t* + 1_), where *S*_*i, t*_ and *S*_*j,t*+1_ represent respectively a cluster size state *i* and *j* at time *t* and *t* + 1.

The MSM describes the dynamics among the cluster states using a transition matrix ***P***, where its element ***P***(*i*, *j*) is the transition’s probability for a peptide, currently in state *i* (*i*-mer), to move to state *j* (*j*-mer) in the next step. The transition probability ***P*** is calculated by normalizing the transition matrix elements with the sum of elements contained in the corresponding row as follows: ***P***(*S*_*i*_, *S*_*j*_) = ***T***(*S*_*i*_, *S*_*j*_) / ∑_*j* = 1_^27^ (*S*_*i*,*t*_ → *S*_*j*,*t* + 1_). The fraction of trajectories in each state (*N*-mer) after *n* propagation steps is thus computed as the row vector *π* (*n*) = *π* (0) ***P***^*n*^, where *π* (0) is a row vector containing the starting fractional populations.

## Results and Discussion

### Cluster Analysis

**Figure 1:**
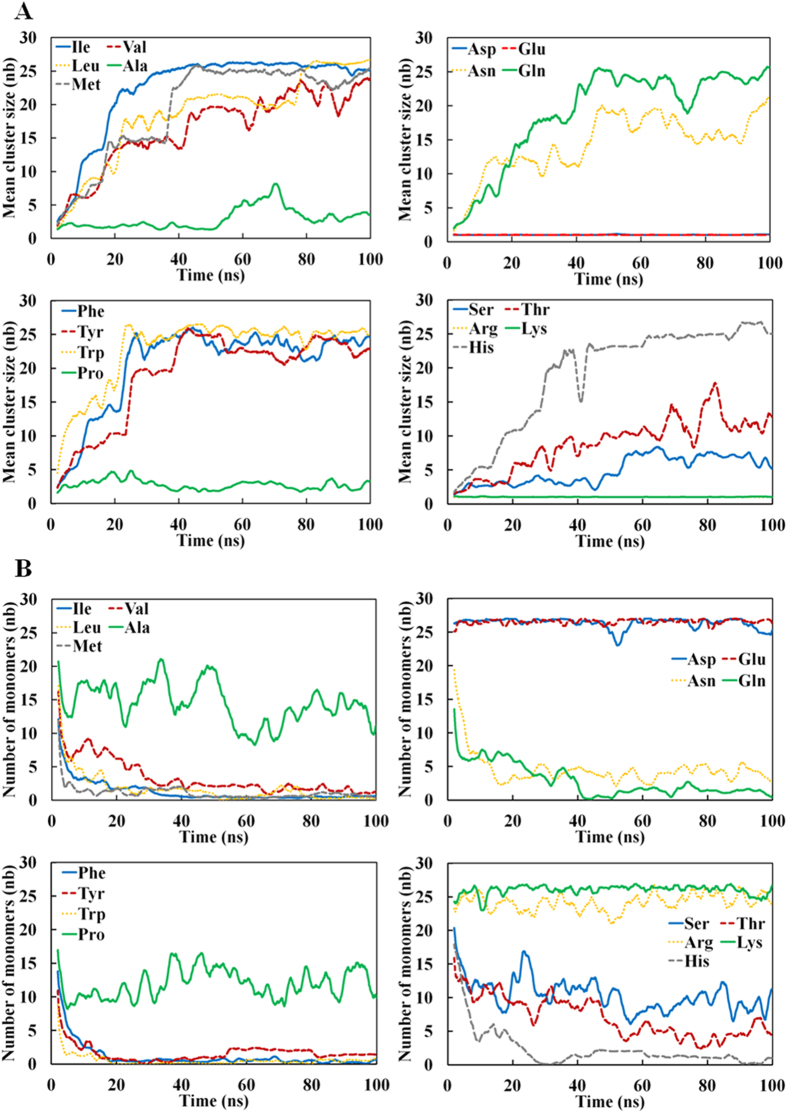
Time-dependence of the mean cluster size (*MCS*) and number of monomer **(B)** for 18 simulated systems.(**A**) *MCS* and (**B**) the number of monomers are, respectively, the mean number of peptides forming cluster and the number of monomeric peptides, as defined using the default cutoff parameters given in the Method section.

**Figure 2:**
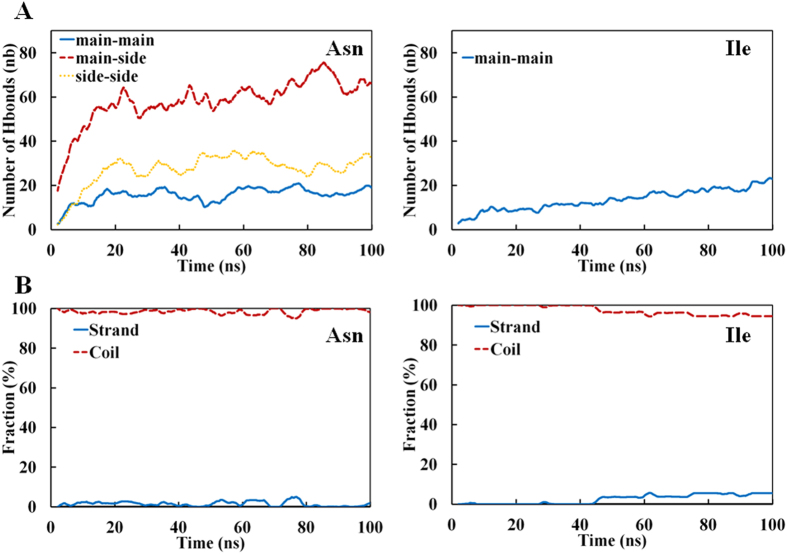
Time-dependent number of inter-peptide H-bonds formed (**A**) H-bonds between mainchains of Ile4 and Asn4. H-bonds between mainchain and sidechain, and sidechain and sidechain are also shown for Asn4 (**A**). (**B**) Secondary structures, i.e. coil, strand and helix, formed in Ile4 and Asn4 as calculated using DSSP. The helix faction overlaps with the bottom horizontal axis and is not visible.

We analyzed the formation of clusters during the MD trajectories, as we anticipated that the cluster’s size could be related to the solubility (or aggregation propensity) of an amino acid. From the 100 ns MD simulations, we found that the tetra-peptides made of I, V, L, N, Q, F, M, H, W, and Y formed large size amorphous clusters, which consisted of 23 ~ 27 peptides (85 ~ 100% of all peptides in the system), whereas those containing E, D, R, and K did not cluster at all (Fig. 1 and Table 1, Fig. S2). The tetra-peptide made of T formed medium size amorphous clusters, consisting of ≤20 peptides (less than 74% of all peptides), and tetra-peptides containing A, P, and S formed small clusters containing less than 10 peptides (less than 37% of all peptides). The above observations are generally in line with our expectations drawn from experimental solubility and hydrophobicity data including our own ones^14^,^34^. Similar conclusions were reached using the fraction of monomers (Fig. 1B) or monomers and dimers (Fig. S2), which was expected since the monomer fraction correlates well with the *MCS*. Additionally, we did not observe β sheets or bridges in our clusters (Fig. 2B), as expected since the tetra peptides are not amyloid forming peptides, but perhaps also because the force field do not favor beta- strands formation in contrast with many amyloid MD simulations^19^,^22^,^23^. Overall, the analysis indicated that the results of a full atom molecular dynamics simulation of a multiple peptide system is indeed be related to experimentally determined “solubility” values.

### Robustness of the Results

**Figure 3:**
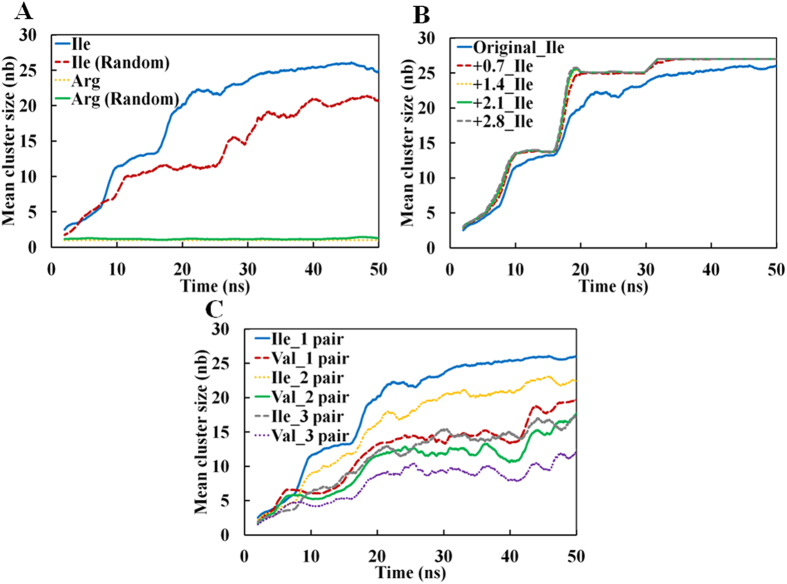
Effect of parameter variation on the *MCS* analysis. Time-dependent *MCS*s obtained from MD simulations starting with a set of 27 tetra-peptides placed randomly (**A**). Though the *MCS* of Ile4 after a 100 ns simulation decreased slightly from 27 to 23, the trajectories clearly indicate that the final size will eventually reach the same value. (**B**) *MCS*s as a function of time calculated by adding 0.7, 1.4, 2.1, and 2.8 Å to the interatomic vdW distance used for identifying a cluster. (**C**) *MCS*s as a function of time calculated by varying the number of atoms pairs for defining a cluster from 1 to 3.

To assess the robustness of our results we first examined the influence of the starting configuration on the results. To this end, we repeated the above calculation with 27 randomly disposed Ile4 and Arg4 peptides. Arginine did not cluster in agreement to the above results obtained with peptides disposed at equal intervals on a grid. Similarly, randomly disposed Ile4 clustered in line with our above results (Fig. 3A). Hence, these calculations confirmed that the initial configuration did not significantly influence the aggregation states reported thereafter.

Additionally, we examined possible influence of the initial peptide’s conformation on the aggregation states as reported thereafter. To this end, we performed MD simulations of single tetra-peptides (Ile4 and Arg4) and compared their conformation using the Phi/Psi dihedral angles with those adopted during the initial 10 ns of the 27-tetra-peptides systems (Fig. S3). In brief, the peptides in both the 27-tetra-peptide and isolated tetra-peptide systems adopted, during the initial stage of the simulation, all of the allowed Phi/Psi angle region including the α-helix and the extended (β) structures, strongly suggesting that the initial conformation of the peptides had no or a minimal influence on our calculation.

We next examined the influence of the parameters, namely, the inter-atomic distances and the number of atom pairs, used for defining the clusters. Although the use of stringent cutoff values slightly reduced the cluster sizes, the trends remained the same (Fig. 3B,C), strongly suggesting that the trends are independent from parameters used to define the clusters. In order to investigate why hydrophobic Ala4 did not form a large cluster we increased the number of peptides of Ala4 and Arg4 in the simulation boxes (54 peptides). Ala4 formed a larger cluster at higher concentration (Fig. S4) but Arg4 did not form any cluster. We concluded that, in contrast to the other peptides, the hydrophobicity of Ala4 was observed at higher concentration. We also confirmed the dependency of Ala4 aggregation on peptide-concentration in this result.

### Insights into the Molecular Mechanisms of Cluster Formation

**Figure 4:**
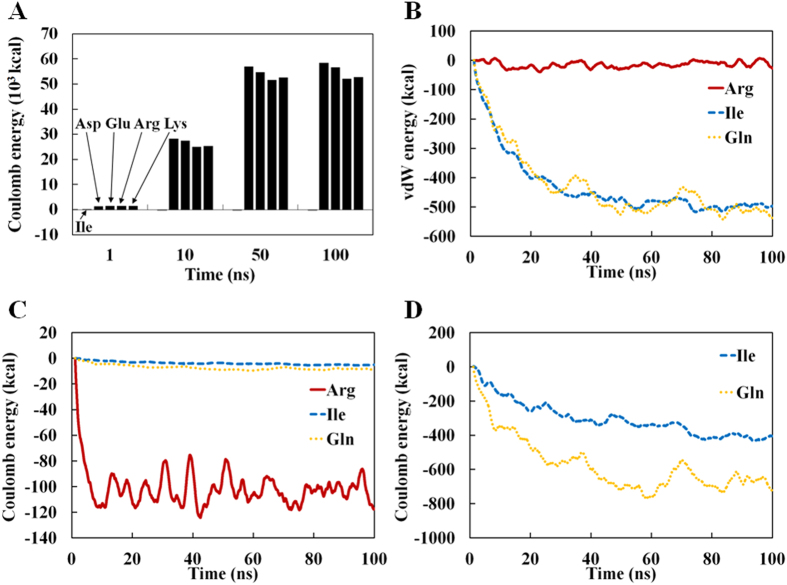
Time-dependent differences of Coulomb and vdW interaction energies for clustered and non-clustered systems of charged residue peptides. (**A**) Coulomb interaction energies (dielectric constant = 1.0) for clustered systems of charged residue peptides; The electrostatic interaction energies of clustered charged peptides, which did not cluster during the MD simulation, were calculated by using the backbone structures of clustered Ile4 peptides and mutating the sidechains to those of the charged amino acids after removal of potential atomic clashes arising from the mutations by energy-minimization. (**B**) vdW energies; and Coulomb interaction energies computed using a dielectric constant of 80.0 (**C**) and 1.0 (**D**).

Repulsive electrostatic interaction appeared to hamper the formation of clusters for charged tetra-peptides (D, E, R, K; Fig. 1A)^14^. This view was confirmed by calculating the Coulomb energies of tetra-peptides in clustered and non-clustered states (Fig. 4A). On the other hand, uncharged amino acids, including polar ones, did not exhibit any repulsive effect as expected. Further, both vdW and Coulomb energies showed good correlation with *MCS* with correlation coefficients of 0.7 ~ 0.9 for systems with *MCS* > 10.0 (Figs S5, S6 and Table S1).

For the purpose of discussion, let us consider the above results with regard to the physical forces that compose a “solvation energy”, which is used to describe a molecule’s solubility and aggregation in thermodynamic terms but are not explicitly included in all-atom MD simulations^25^. Overall, the aforementioned results indicate that both vdW and Coulomb interactions contribute to the clustering of the peptides, but their roles are different. The solubility of charged tetra-peptides originated from inter-molecular repulsive electrostatic forces, whereas no repulsion occurred between neutral amino acids, which cluster through vdW interaction (Fig. 4) and H-bonds (see next paragraph). Noteworthy, the strength of repulsive electrostatic interaction between charged residues is much larger than the vdW interaction stabilizing the clusters of neutral amino acids (Fig. 4B,D). Similarly, polar amino acids clustered through vdW interaction but the clusters disintegrated when the electrostatic interaction between partial charges become destabilizing. Though these observations are in line with one’s intuitive anticipation, this is the first systematic and quantitative analysis of the physical forces that drive the solubility of peptides from an atomistic viewpoint.

We also examined the contribution of H-bonds to the formation of clusters. The mainchains of clustered hydrophobic peptides formed a substantial number of H-bonds (Fig. 2A), which was approximately the same as the number of mainchain-mainchain H-bonds in polar peptides (Fig. 2A). Among polar amino acids, the *MCS* of Thr4 and Ser4 were smaller than those of Gln4 and Asn4 (Fig. 1), but the numbers of mainchain-mainchain H-bonds, both water mediated and non mediated ones, were approximately the same in all four peptides (Table 2). To date, mainchains H-bonds were mostly intermolecular and no secondary structures were formed as assessed by DSSP^35^ (Fig. 2B).

We further examined H-bonds formed by the sidechains of polar amino acids, as they contain electron acceptors and donors for H-bonding. Sidechains H-bonds exhibited different properties among the polar amino acids: Thr4 and Ser4 formed virtually no direct and very few water mediated H-bonds between their sidechains as they have no electron donors (Table 2). Similarly, Thr4 and Ser4 formed fewer direct sidechain-mainchain H-bonds than Gln4 and Asn4, but almost the same number of water mediated sidechain-mainchain H-bonds were observed in all four tetra-peptides (Table 2). All in all, these results are fully in line with the fact that the sidechains of Asn and Gln, which have both a donor and an acceptor, have a stronger ability to form H-bonds than Ser and Thr sidechains, which have only an acceptor.

**Figure 5:**
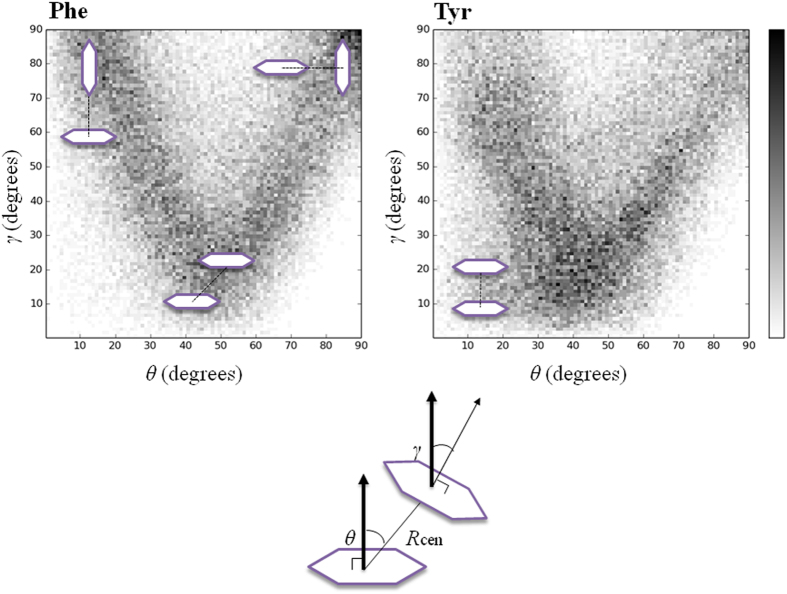
Spacial orientation of closely located aromatic rings. The distribution of *θ* and *γ* angle for pairs with *R*_*cen*_ < 5.5 Å were calculated for Phe4 and Tyr4. A schematic explanation of the parameters (*R*_*cen*, _*θ* and *γ*) describing the ring’s conformation is given at the bottom of the figure. *R*_*cen*_ is the distance between the two geometrical centers of the aromatic rings. *θ* is the angle between the normal vector to the first ring and the vector relying the two ring centers. *γ* is the angle between the two ring’s surface normal vectors.

Tetra-peptides made of aromatic residues (Y, W, F) formed large clusters (Fig. 1), where several π − π interactions were present (Fig. S7A). Tyrosine’s hydroxyl groups (−O_η_H) were mostly solvent exposed and formed H-bonds with water molecules, but a few formed a H-bond with a mainchain’s carbonyl group or a -O_η_H (Figs. S7B,C). Comparison with Phe4 indicated that the presence of −O_η_H caused only a minor solubility change (Fig. 1), as assessed both by *MCS* and the fraction of monomers. Although many CH-π stackings were observed in both Tyr4 and Phe4 clusters, face-to-face stacking were present only in Tyr4, not in Phe4 clusters, possibly due to the presence of −O_η_H in Tyr (Fig. 5). Thus, the phenol ring adopted specific configurations including π- and T- stacking when the rings were close to each other, which is generally in line with previous simulation and PDB database analysis^36^.

### Buried Water Molecules

As mentioned above, several buried peptide groups formed H-bonds, some of which were formed with buried water molecules, which motivated us to further characterize them. We defined buried water as molecules located within 75% of the radius of gyration from the center of gravity. Buried water molecules were observed in all tetra-peptide clusters, including Thr4 and Ser4’s clusters despite their smaller *MCS*. Clusters of peptides composed of hydrophilic residues tended to contain more buried water molecules than those made of hydrophobic amino acids. Peptide clusters made of aromatic amino acids had high water contents, and among all peptides Tyr4 had the highest one (Table 3).

### Relationship between Experimental Solubility/Hydrophobicity scales and Cluster Formation

**Figure 6:**
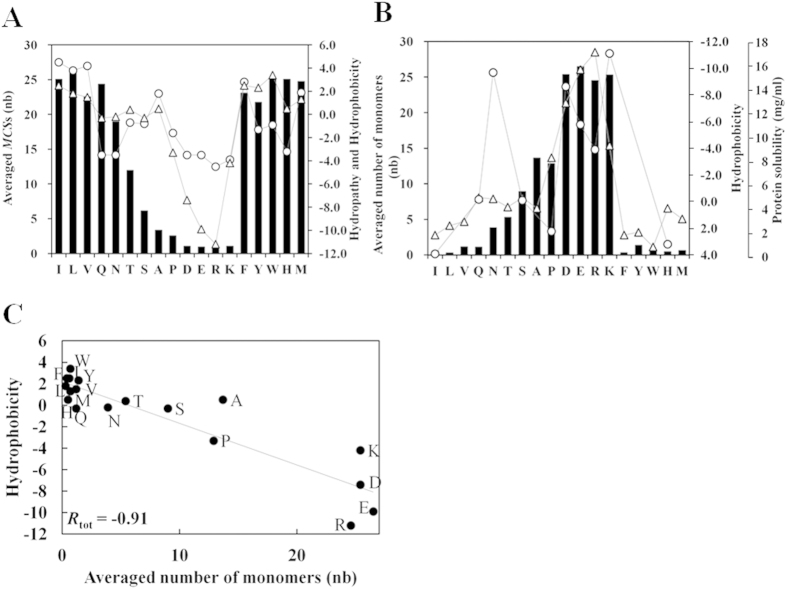
Comparison of calculated solubility parameters and experimental solubility/hydrophobicity scales. In both panels, the calculated parameters are represented by black bars. (**A**) Averaged mean cluster size (black bar) vs hydropathy (circle)^8^ and hydrophobicity (triangle)^41^,^42^. The number of monomers and mean cluster sizes are values averaged over the last 10 ns of the MD simulations. (**B**) The number of monomer (black bar) averaged over the last 10 ns is shown vs hydrophobicity (triangle) and the contribution to protein solubility (circle) that we reported in an earlier study^14^. (**C**) Correlation between the averaged number of monomer and hydrophobicity. *R*_tot_ indicates the correlation coefficients of all residues.

Our calculation uses force field derived from physico-chemical first principles. Aggregation or low solubility is usually considered to be caused by the so called “hydrophobic” interactions^37^, which, in our calculation originates from the absence of repulsive electrostatic forces, the absence of H-bonds with water molecules, the formation of inter-peptide H bonds, and the occurrence of attractive short range vdW interaction as discussed in the above subsection. We thus assessed potential correlation between our results and selected experimental hydrophobicity/solubility scales (Figs 6 and S8). The relative amino acid’s solubility and hydrophobicity were overall well reproduced in our calculation. Namely, hydrophobic and aromatic residues formed clusters and the *MCS* ranked as A ≪ V < Y < F~W~I~L which is roughly reflected in the hydrophobicity scales (A < V < L < Y~I~F < W; Table 1). Similarly, the *MCS* of polar tetra-peptide and Met4 ranked as S < T < N < Q ≪ M which also reflected the ranking of their hydrophobicities (S~N~Q < T ≪ M). Finally charged residues (R, D, E, K) remained monomeric (*MCS* ~ 1) which would correspond to their high solubilities or low hydrophobicities. Similar patterns of correlations were observed with the hydropathy scale, which correlates strongly correlate to hydrophobicity (Table 1). Finally, the present results roughly reproduced a recent amino acid’s hydrophilicity scale as well as a recently determined amino acid contribution scale to proteins’ solubility (Fig. S8; *R* = 0.87 calculated without Asn).

The lowest correlation was found with the individual amino acid’s solubilities as tabulated in the CRC Handbook of Chemistry and Physics (*R* = −0.38 excluding Pro)^9^, where proline has a reported solubility of 1600 g/L and Lysine’s solubility is low at 5 g/L. These two figures, which are not reproduced in our calculation, contradict our intuitive anticipation and are also at odd with several solubility/hydrophobicity scales (Table 1). Thus, given such moderate correlation among the various experimental solubility and hydrophobicity scales, correlation coefficients of up to ~0.9 between the fraction of monomer as well as the *MCS* and some of the experimental scales is surprisingly good, and one may hope that they could serve as a scale for estimating an amino acid’s contribution to a peptide or protein solubility.

### MSM Analysis

**Figure 7:**
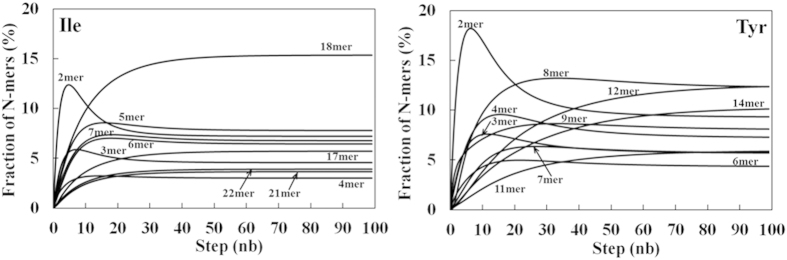
Markov State Model analysis of *N*-mer fraction computed by propagating state (*N*-mer) transition probabilities using the MSM. The top ten higher fractions of *N*-mer are shown for representative tetra-peptides.

We analyzed the MD trajectories using MSM, which provides a convenient way to model kinetic networks between different conformational states^38^,^39^. MSM is appropriate for analyzing the MD results because of the relatively large size of our 27-peptide system, which was not the case for much smaller systems used in previous studies^22^,^23^. We calculated the transition matrix ***P*** whose components are the fraction of *i*-mers undergoing a transition to a *j*-mer at a given propagation step. In order to analyze the initial stage for the aggregation mechanism, we used the first 20 ns trajectories for constructing the transition matrix. MSM revealed that small clusters (2~4-mer) rapidly accumulated, with dimmers constituting up to 18% of the oligomers (Fig. 7; Tyr), before growing or consolidating into medium (5~9-mer) and large size amorphous clusters (greater than 10-mer). The small size clusters might correspond to seeds playing a key role at the initial stage of amorphous aggregation^18^,^40^.

## Conclusion

We reported a systematic and in-depth analysis of amino acid’s contribution to protein/peptide solubility using a molecular dynamics simulation of multi-peptide systems with explicit solvents. Eighteen 100 ns MD simulations of tetra-peptides corresponding to all amino acids, but Gly and Cys, were carried out. The results were overall in line with previously reported experimental amino acid’s solubility and hydrophobicity scales. To our knowledge, this is the very first study of this kind, and it was surprising that the solubility of amino acids is fairly well reproduced using standard MD simulation methods, without a need to introduce artificial attractive or repulsive forces. A finer analysis of the calculation indicated that the high solubility of charged residues originated from repulsive Coulomb energies, whereas the lower solubility of uncharged residues had various origins. To date, “hydrophobic” residues, such as Ile, Leu and Val, which lack repulsive electrostatic interaction, clustered predominantly through vdW interactions and accessorily through mainchain H-bonds. Markov state model analysis suggested that small clusters consisting of 2 ~ 4 peptides accumulated before growing or merging into larger clusters.

## Additional Information

**How to cite this article**: Kuroda, Y. *et al.* All-atom molecular dynamics analysis of multi-peptide systems reproduces peptide solubility in line with experimental observations. *Sci. Rep.* **6**, 19479; doi: 10.1038/srep19479 (2016).

## Supplementary Material

Supplementary Information

## Figures and Tables

**Table 1:** Averaged mean cluster size and solubility/hydrophobicity scales.

Amino acid residues	Parameters						
	Averaged *MCS*^a^	Individual amino acid solubility^b^ (g/kg)	Hydropathy^c^	Hydrophilicity^d^ (kcal/mol)	Hydrophobicity^e^ (kcal/mol)	Protein solubility^f^ (mg/ml)	Averaged number of monomers
Ile	25.1	34.2	4.5	0.2	2.5	0.1	0.6
Leu	26.2	23.8	3.8	0.1	1.8	Na	0.3
Val	22.6	88.0	4.2	0.4	1.5	Na	1.2
Gln	24.4	42.0	−3.5	11.8	−0.3	4.7	1.2
Asn	19.0	25.1	−3.5	12.1	−0.2	15.4	3.9
Thr	12.0	90.6	−0.7	7.3	0.4	Na	5.4
Ser	6.2	250.0	−0.8	7.5	−0.3	4.6	9.0
Ala	3.4	166.9	1.8	0.5	0.5	Na	13.7
Pro	2.6	1625	−1.6		−3.3	2.0	12.9
Asp	1.1	5.4	−3.5	13.3	−7.4	14.2	25.4
Glu	1.0	8.6	−3.5	12.6	−9.9	11.0	26.5
Arg	1.0	182.6	−4.5	22.3	−11.2	8.9	24.6
Lys	1.1	5.8	−3.9	11.9	−4.2	17.0	25.4
Phe	23.1	27.9	2.8	3.2	2.5	Na	0.4
Tyr	21.8	0.5	−1.3	8.5	2.3	Na	1.4
Trp	24.8	13.2	−0.9	8.3	3.4	Na	0.7
His	25.1	43.5	−3.2	12.6	0.5	0.9	0.5
Met	24.8	56.0	1.9	3.9	1.3	na	0.7
	*R*^g^	−0.35 (−0.38)	0.51	−0.47	0.79	−0.50	−0.93
	*R*^h^	0.13 (0.13)	−0.58	0.58	−0.91	0.64	—

^a^*MCS*s and number of monomers averaged over the last 10 ns of simulation.

^b^Ref. 9.

^c^Ref. 43.

^d^Ref. 41,42.

^e^Ref. 44,45.

^f^Ref. 14.

^g^Correlation coefficient *R* between *MCS* and various parameters.

^h^Correlation coefficient *R* between averaged number of monomers and various parameters.

**Table 2:** Averaged number of buried H-bonds per residue for polar amino acids.

		*MCS*^a^	P-P^b^	P-W^c^	P-W-P^d^
SC-SC^e^	Asn	19.0	0.28	1.96	0.32
	Gln	24.4	0.27	1.98	0.38
	Ser	6.2	0.07	1.95	0.09
	Thr	12.0	0.07	1.44	0.21
MC-MC^f^	Asn		0.16	1.30	0.21
	Gln		0.16	1.18	0.22
	Ser		0.17	1.66	0.23
	Thr		0.18	1.32	0.21
SC-MC	Asn		0.60		0.50
	Gln		0.56		0.43
	Ser		0.19		0.44
	Thr		0.21		0.45

^a^*MCS* are averaged over last 10 ns.

^b^Number of H-bonds formed between peptides.

^c^Number of H-bonds formed between peptide and water molecule.

^e^Number of water-mediated H-bonds formed between peptides.

^f^SC stands for sidechain.

^g^MC stands for mainchain.

**Table 3:** Number of buried water molecules, *MCS*, and the radius of gyration (*Rg*) averaged over 50 ~ 100 ns of the MD simulations.

	Number of water^a^	*MCS*^b^	*Rg* (Å)^c^
Ile	11.2	26.5	13.3
Leu	15.1	25.9	14.5
Val	13.6	22.7	14.7
Met	11.6	25.5	14.2
Asn	27.8	20.8	14.4
Gln	39.9	25.2	15.6
Ser	21.3	14.0	11.3
Thr	33.2	16.8	13.9
Phe	39.5	24.4	16.7
Tyr	162.7	24.5	19.7
Trp	81.8	25.0	18.4

^a^Water molecules buried into the clusters.

^b^Mean cluster size.

^c^Radius of gyration of the clusters.
