# Supplementary material for: All-atom molecular dynamics analysis of multi-peptide systems reproduces peptide solubility in line with experimental observations
Source: Sci Rep. 2016 Jan 28;6:19479. doi: 10.1038/srep19479 (PMC4730209; doi:10.1038/srep19479)
Supplement: Supplementary Information [file srep19479-s1.doc]

Supporting Information

**All-atom molecular dynamics analysis of multi-peptide systems reproduces peptide solubility in line with experimental observations**

Yutaka Kuroda^$,1*^, Atsushi Suenaga^$,2^, Yuji Sato^$,1^, Satoshi Kosuda^1^, Makoto Taiji^3^

^1^ Department of Biotechnology and Life Sciences, Graduate School of Engineering, Tokyo University of Agriculture and Technology, Koganei-shi, Nakamachi, Tokyo 184-8588, Japan

^2^ Molecular Profiling Research Center for Drug Discovery (molprof), National Institute of Advanced Industrial Science and Technology (AIST), 2-4-7 Aomi, Koto-ku, Tokyo 135-0064, Japan

^3^ Computational Biology Research Core, Quantitative Biology Center (QBiC), RIKEN, IMDA Building (6F), 1-6-5 Minatojima-Minami-machi, Chuo-ku, Kobe, Hyogo 630-0047, Japan

^$^ Equal contribution

^*^Correspondence to Y. K. E-mail: ykuroda@cc.tuat.ac.jp, Phone: +81-422-388-7794

Figure S1.

Graphical representation of the Initial configuration of the 27 tetra-peptides. The peptide conformations are initially extended but they quickly adopt all possible conformation after 10 ns, which is before oligomerization starts (See main text and Fig S9). but The cube’s dimensions are approximately 104×104×104 Å^3^. Water molecules are not shown.


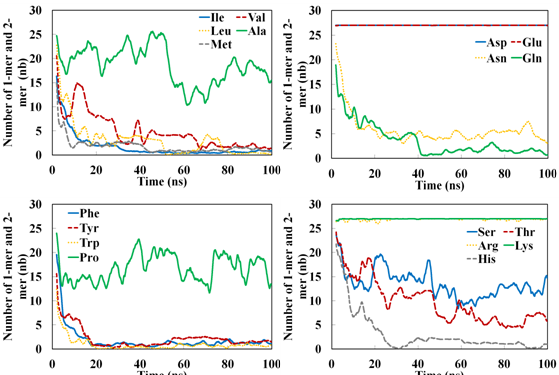


Figure S2.

Time-dependent number of monomer and dimer for 18 simulated systems.


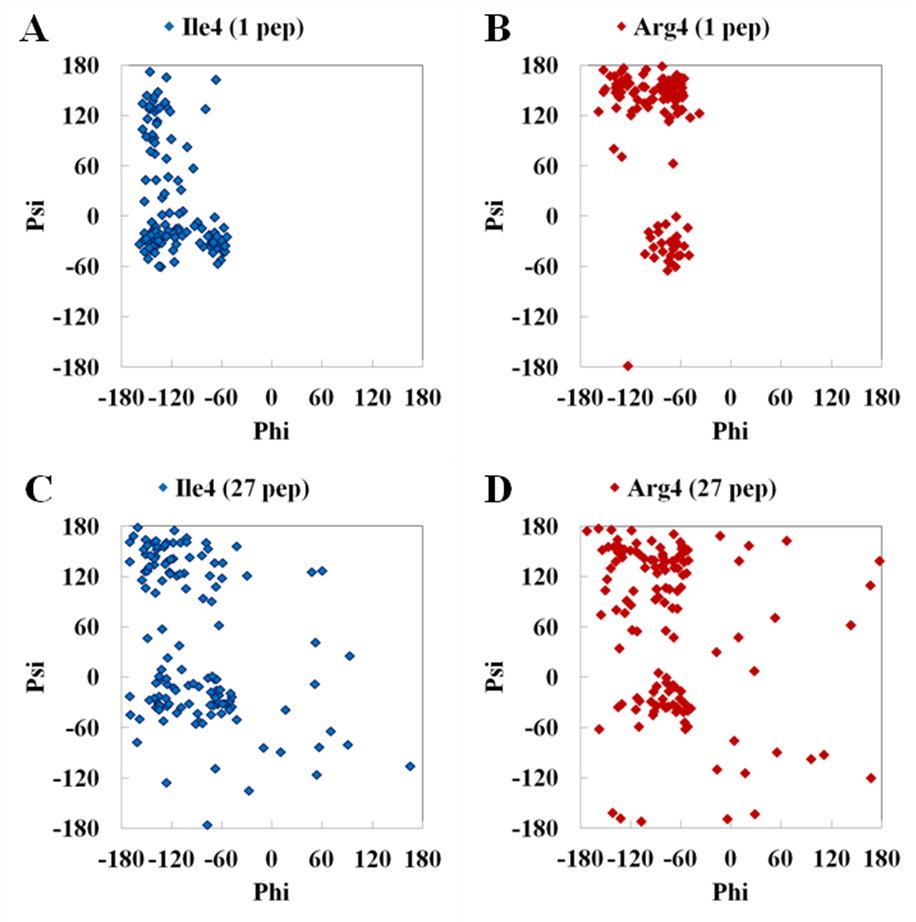


Figure S3.

Ramachandran plot of Phi/Psi dihedral angles. In A and B, we analyzed the trajectories of isolated single-peptide (Ile4 and Arg4) simulations between 9.7 to 10.0 ns. In C and D, we analyzed the trajectories of 27 peptides of Ile4 and Arg4 at 10 ns.


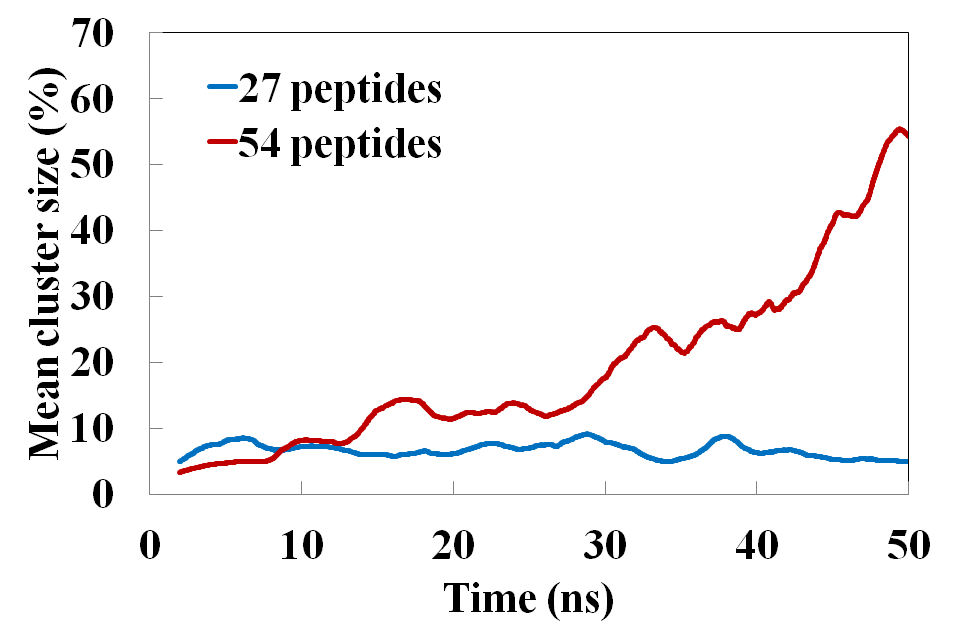


Figure S4.

Trajectories of the mean cluster size for systems containing respectively 27 and 54 Ala4-peptides. All conditions are the same in both calculations except for the number of peptides included in the systems.


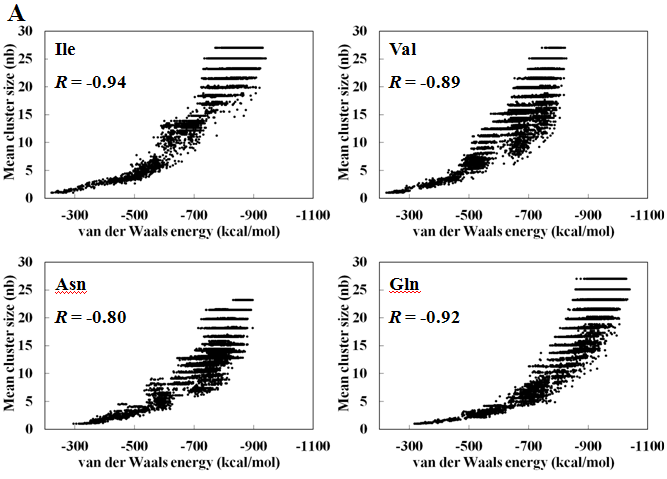


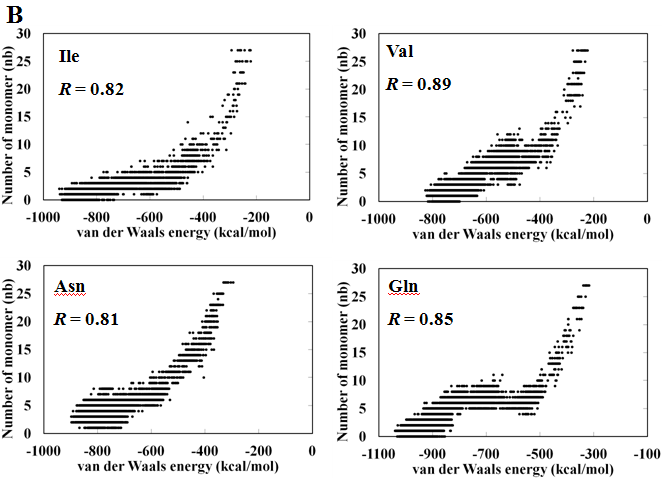


Figure S5.

Correlation between van der Waals energies and mean cluster size (A) or the number of monomers (B). *R* indicate the correlation coefficients.


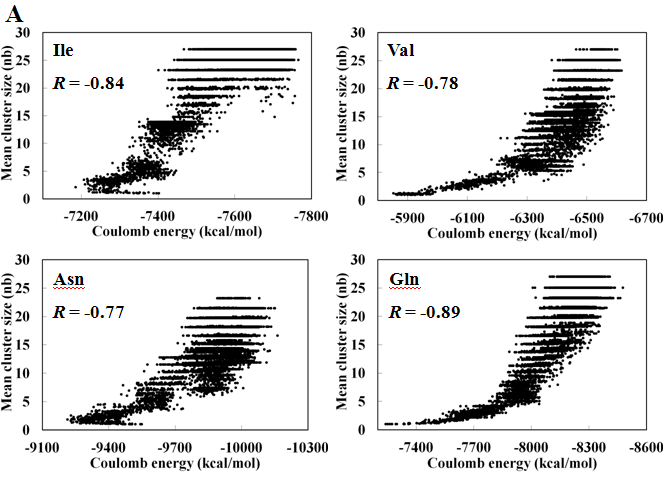


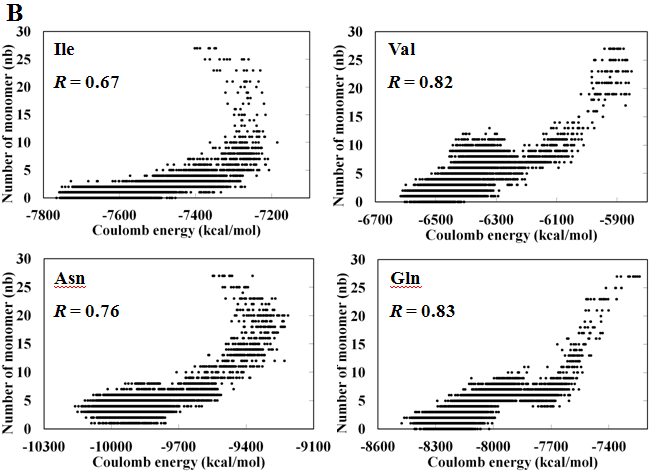


Figure S6.

Correlation between Coulomb energies and mean cluster size (A) or the number of monomers (B). *R* indicate the correlation coefficients.


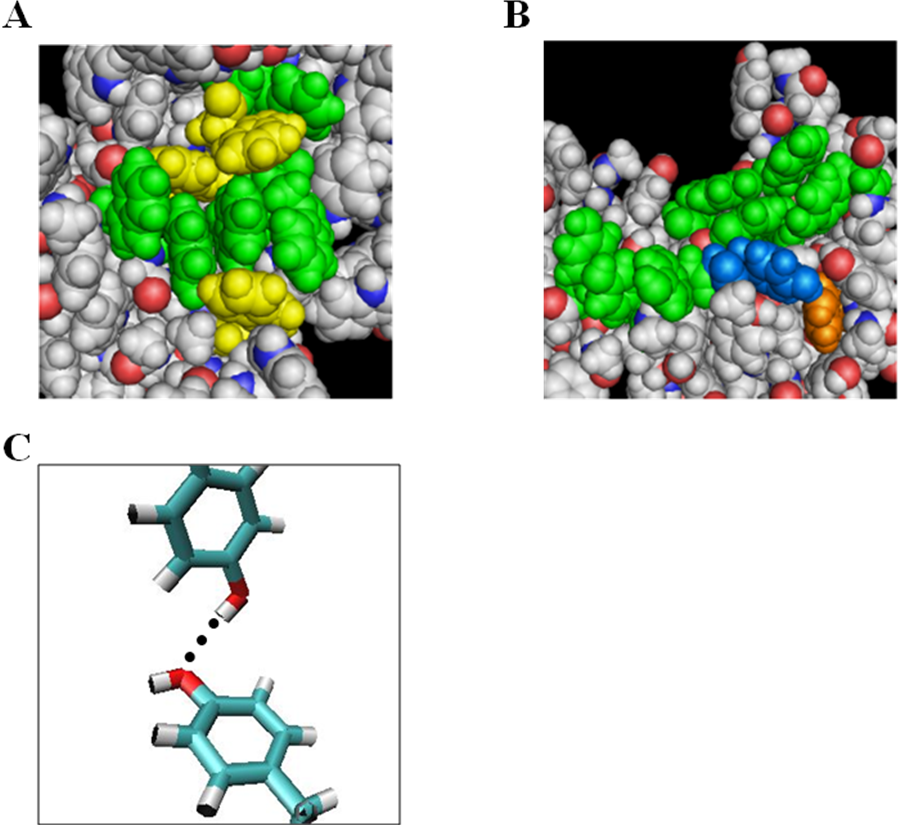


Figure S7.

Inter- and intra-strand interactions of (A) Trp4, and (B and C) Tyr4 systems. (A) Trp sidechains are colored green and yellow. Green and yellow aromatic rings form CH-π interactions, whereas green aromatic rings interact with each other through face-to-face stacking. (B) Green color coding is the same as in (A). Blue and orange aromatic rings are hydrogen bonded through their –O_η_H groups. (C) A hydrogen bond between two –O_η_H is shown as a dotted line.


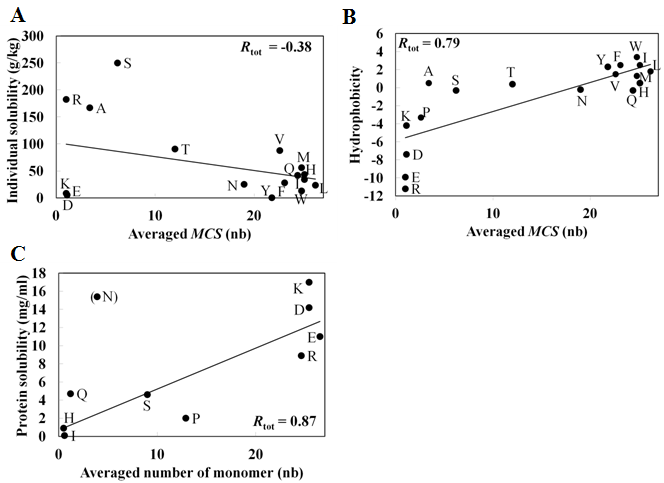


Figure S8.

Correlation between averaged mean cluster size (*MCS*) and (A) individual amino acid solubility, (B) hydrophobicity. Correlation between averaged number of monomer and (C) protein solubility. *R*s indicate the correlation coefficients of all residues. In C, the solubility of Asn tagged protein was removed from the calculation of the correlation coefficient.

**Table S1. Correlation coefficients (*R*) between *MCS* and vdW/Coulomb energy**

| Amino acid residues | Averaged *MCS*^a^ | *R* (vdW)^b^ | *R* (Coulomb)^c^ |
| --- | --- | --- | --- |
| Ile | 25.1 | -0.94 | -0.84 |
| Leu | 26.2 |  |  |
| Val | 22.6 | -0.89 | -0.78 |
| Asn | 19.0 | -0.81 | -0.77 |
| Gln | 24.4 | -0.92 | -0.9 |
| Ser | 6.2 | -0.87 | -0.64 |
| Thr | 12.0 | -0.87 | -0.71 |
| Ala | 3.4 | -0.87 | -0.57 |
| Arg | 1.0 |  |  |
| Asp | 1.1 |  |  |
| Glu | 1.0 |  |  |
| Lys | 1.1 |  |  |
| Phe | 23.1 | -0.87 | -0.63 |
| Tyr | 21.8 | -0.87 | -0.86 |
| Trp | 24.8 | -0.88 | -0.8 |
| His | 25.1 | -0.92 | -0.91 |
| Pro | 2.6 | -0.75 | -0.40 |
| Met | 24.8 | -0.90 | -0.87 |

^a^ *MCS*s averaged over the last 10 ns of simulation.

^b^ Correlation coefficient *R* between *MCS* and van deer Waals energy.

^c^ Correlation coefficient *R* between *MCS* and Coulomb energy.

**Table S2. Correlation coefficients (*R*) among computational and experimental values. Values in parentheses are *R* without proline.**

|  | Averaged *MCS*^a^ | Averaged number of monomer | Individual amino acid solubility^b^ | Hydropathy^c^ | Hydrophilicity^d^ | Hydrophobicity^e^ |
| --- | --- | --- | --- | --- | --- | --- |
| Averaged Number of monomer | -0.92 |  |  |  |  |  |
| Individual amino acid solubility | -0.35 (-0.38) | 0.13 (0.13) |  |  |  |  |
| Hydropathy | 0.51 | -0.48 | -0.06 (0.06) |  |  |  |
| Hydrophilicity | -0.48 | 0.54 | 0.03 | -0.93 |  |  |
| Hydrophobicity | 0.79 | -0.91 | -0.15  (-0.13) | 0.65 | -0.75 |  |
| Protein solubility^f^ | -0.51 | 0.87 | -0.32 | -0.60 | 0.43 | -0.49 |

^a^ *MCS*s are averaged over last 10 ns.

^b^ Ref .

^c^ Ref. .

^d^ Ref. and

^e^ Ref. and .

^f^ Ref .
